# Impact of High-Intensity Statin on Early Neurologic Deterioration in Patients with Single Small Subcortical Infarction

**DOI:** 10.3390/jcm12093260

**Published:** 2023-05-03

**Authors:** Seong Hwa Jang, Hyungjong Park, Jeong-Ho Hong, Joonsang Yoo, Hyung Lee, Hyun Ah Kim, Sung-Il Sohn

**Affiliations:** 1 Department of Neurology, Keimyung University School of Medicine, Daegu 42601, Republic of Korea; seonghwajang0416@gmail.com (S.H.J.); neurohong79@gmail.com (J.-H.H.); hlee@dsmc.or.kr (H.L.); kha0206@dsmc.or.kr (H.A.K.); sungil.sohn@gmail.com (S.-I.S.); 2 Department of Neurology, Yonsei University College of Medicine, Yongin Severance Hospital, Yongin 16995, Republic of Korea; quarksea@gmail.com

**Keywords:** early neurologic deterioration, single small subcortical infarction, statin, atherosclerosis, ischemic stroke

## Abstract

Backgrounds: One of the major hypotheses for early neurological deterioration (END) in single small subcortical infarction (SSSI) is the process of atherosclerosis. However, the association between statin therapy, especially high-intensity statin therapy, and its effectiveness in reducing the incidence of END during the acute phase of SSSI remains unclear. This study aimed to investigate the influence of high-intensity statin therapy compared to moderate-intensity statin therapy during the acute phase on the incidence of END in SSSI. Methods: The records of 492 patients with SSSI who received statin therapy within 72 h of symptom onset from a prospective stroke registry were analyzed. The association between END and statin intensity was evaluated using multivariable regression analysis for adjusted odds ratio (aOR). Results: Of the 492 patients with SSSI (mean age: 67.2 years, median NIHSS score on admission: 3), END occurred in 102 (20.7%). Older age (aOR, 1.02; 95% confidence interval (CI), 1.00–1.05; *p* = 0.017), and branch atheromatous lesion (aOR, 3.49; 95% CI 2.16–5.74; *p* < 0.001) were associated with END. Early high-intensity statin therapy was associated with a lower incidence of END than moderate-intensity statin therapy (aOR, 0.44; 95% CI, 0.25–0.77; *p* = 0.004). In addition, there was significantly lower incidence of END in early administration (≤24 h) of high-intensity statin group. Conclusions: We identified an association between the intensity of early statin therapy and END in patients with SSSI. Early administration of high-intensity statin (≤24 h) is associated with a reduced incidence of END in patients with SSSI.

## 1. Introduction

Single small subcortical infarction (SSSI), also called lacunar infarction, constitutes approximately 20% of all ischemic stroke subtypes [1]. Traditionally, SSSI is caused by the occlusion of deep perforating arteries, mainly pathologically characterized by lipohyalinosis or fibrinoid degeneration [2]. However, in some patients, localized atherosclerotic processes, such as branch atheromatous lesions, have been implicated in SSSI development due to microatheroma or junctional atherosclerotic plaque [3].

The outcome of SSSI is relatively good; however, approximately 17% of patients experience early neurologic deterioration (END) after SSSI, which is associated with unfavorable outcomes [4]. To date, intervention for preventing and treating END in SSSI has not been established. Therefore, it is crucial to investigate the potential mechanism to identify a treatment strategy to prevent END.

There is growing evidence that the atherosclerotic process may be a major mechanism of END [3,5]. Similarly, there is evidence of the efficacy of statin therapy in patients with atherosclerosis. High-intensity statin therapy is associated with a reduction in atherosclerosis progression compared with moderate-intensity statin therapy in patients with coronary artery disease [6]. In the Stroke Prevention by Aggressive Reduction in Cholesterol Levels trial (a stroke-specific trial), high-intensity statin therapy was associated with a reduced risk of recurrent ischemic stroke and cardiovascular events [7]. According to this trial, current guidelines for the secondary prevention of stroke suggest the application of high-intensity statin therapy in patients with atherosclerosis [8]. 

Therefore, we hypothesized that if the atherosclerotic process contributes to the development of END in SSSI, administration of a high-intensity statin, an approved treatment for secondary prevention of ischemic stroke of atherosclerotic origin, may be effective for preventing END in SSSI. This study investigates the impact of high-intensity statin therapy on END prevention in patients with SSSI. 

## 2. Materials and Methods

### 2.1. Study Population

This was a retrospective observational study of prospective registered patients with ischemic stroke in the stroke registry between May 2014 and December 2020. The cohort in this study comprised consecutive patients with acute ischemic stroke who were admitted to the Keimyung University Dongsan Hospital in Daegu, South Korea, within seven days of the onset of their symptoms.

During hospitalization, all patients underwent brain magnetic resonance imaging (MRI) and were managed through a standardized care pathway according to current guidelines. Systemic evaluation, including 12-lead electrocardiography, chest radiography, standard blood test, and lipid profile, was performed. To detect the cardiac embolic source, 24 h Holter monitoring or electrocardiographic monitoring at the stroke unit was performed. Echocardiography was performed to evaluate the presence of structural cardiac abnormalities or evidence of cardiac thrombi. 

### 2.2. Clinical Data Collection

Data were collected on demographics, vascular risk factors, previous history of stroke or transient ischemic stroke, coronary artery disease, and medication history, including antithrombotic drugs, antihypertensive drugs, and statins. The National Institutes of Health Stroke Scale (NIHSS) was used to assess stroke severity. The first administration of statins and their doses and types were retrospectively collected using a web-based data system and electronic medical records. 

### 2.3. Follow-Up and Outcomes

After discharge, the patients were regularly followed up at 3 months, 1 year, and every year thereafter. At each follow-up visit, the modified Rankin scale (mRS) via face-to-face interviews with a neurologist or clinical research associate was used to obtain information in an outpatient clinic. If patients missed a follow-up visit, we obtained information from the patients or their families via telephone interviews based on a structured questionnaire. A favorable outcome was defined as 0–2 on the mRS at 3 months.

### 2.4. Imaging Analysis

All patients underwent 1.5 or 3 Tesla scanner MRI and magnetic resonance angiography (MRA), including cerebral arteries and carotid artery, within 24 h of admission (MRI, Signa VH/I, GE Healthcare, Milwaukee, WI, USA). The scanner parameters for diffusion-weighted imaging (DWI) were as follows: repetition time, 7500 ms; echo time 84 ms, matrix number 128 × 128, 2 b value, 0 and 1000 s/mm^2^; slice thickness, 5 mm; and inter-slice gap, 2 mm. 

SSSI was defined as single ischemic lesions with an axial diameter ≤2 cm with a patent relevant artery (stenosis degree ≤ 50%) without atrial fibrillation in the striatocapsular, thalamic, or brain stem area [5]. 

Branch atheromatous lesion was defined as acute infarction on DWI in 4-axial consecutive MRI cuts at a slice thickness of 5 mm in the lenticulostriate artery territory or infarction extending to the basal surface of the pons [9]. Parent artery stenosis was defined as the presence of ≤50% stenosis in the adjacent artery corresponding to infarction lesion. Two trained neurologists independently reviewed radiologic imaging (k-value: 0.92 for branch atheromatous lesion and 0.88 for parent artery stenosis). Discrepancies were resolved by consensus between the two raters, who remained blinded to the patient’s clinical information. 

### 2.5. Definition of END and Collection

END was prospectively collected from May 2014. END was defined as any new neurologic symptoms/signs or neurologic worsening occurring within 21 days of symptom onset. Any new neurologic symptoms/signs or neurologic worsening satisfy one or more of the following criteria: (1) an increase of ≥2 points in total NIHSS score, (2) an increase of ≥1 point in the motor NIHSS score, and/or (3) an increase of ≥1 point in the consciousness score [10,11,12]. 

The cause of END was categorized as stroke recurrence, stroke progression, symptomatic hemorrhagic transformation, others, and unknown, and this information was collected. The detailed definitions about the cause of END are presented in Methods in the Supplementary Materials [5]. 

The initial NIHSS score at admission was checked daily until hospital discharge. END was immediately reported after its development to the staff on duty or to neurology residents. All END cases were assessed and discussed by all stroke team members in the monthly quality improvement meeting of the stroke team.

### 2.6. Statin Therapy Protocol

Statins were administered within 72 h of symptom onset. The same dose of statins was maintained for at least 21 days after symptom onset. Statins were categorized into moderate- and high-intensity statins. High-intensity statin was defined as dose expected to reduced low-density lipoprotein cholesterol (LDL-c) by greater than or equal to 50%, and moderate-intensity statin was defined as dose expected to reduced LDL-c by 30–50% according to previous guidelines [13,14]. 

### 2.7. Statistical Analysis

Baseline characteristics were described using counts (*n*) and percentages (%) for categorical variables and medians with interquartile ranges for continuous variables. The chi-square test or Fisher’s exact test was performed for categorical variables, and the Mann–Whitney U-test or t-test was used for continuous variables. Multivariable logistic regression analysis was performed to identify the association between END and statin intensity. Variables with *p*-values of <0.2 in the univariable analysis were included to evaluate the independent factors for END in the multivariable logistic regression analysis. In addition, the association of END with favorable outcomes at 3 months was analyzed using multivariable logistic regression analysis adjusting for variables with *p*-values of <0.2 in the univariable analysis. In addition, propensity score matching analysis as sensitivity analysis was performed. The propensity score was calculated using age, sex, initial NIHSS score, and the presence of branch atheromatous lesion. After estimating the propensity score, the high-intensity statin and moderate-intensity statin group were matched at a ratio of 2:1. All analyses were conducted using R version 3.5.1 (R Development Core Team, Vienna, Austria). *p*-values of <0.05 were considered statistically significant.

## 3. Results

### 3.1. Baseline Characteristics

A total of 3988 patients with ischemic stroke within 7 days from symptom onset were registered in the Registry (redacted registry name) between May 2014 and December 2020. From this initial cohort, patients with single subcortical infarction were included (*n* = 1136). We excluded 286 patients: those without MRI or MRA (*n* = 37), with an ischemic lesion of maximal diameter >2 cm (*n* = 73), with underlying stenosis >50% in the relevant artery (*n* = 105), with a cardioembolic source (*n* = 50), and having other determined etiologies (*n* = 21). 

**Figure 1:**
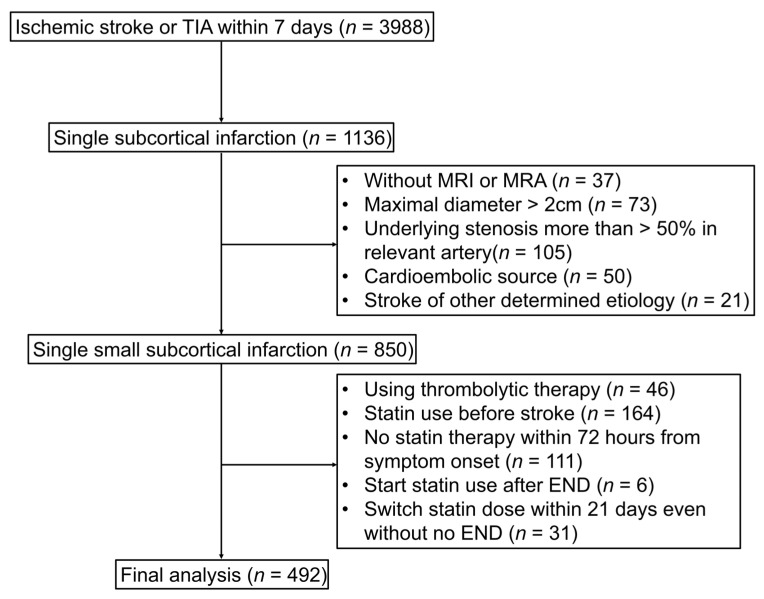
Study flow chart. TIA indicates transient ischemic attack; MRI, magnetic resonance imaging; MRA, magnetic resonance angiography; END, early neurologic deterioration.

Additionally, for the statin dose effect for END to be administered as accurately as possible, patients who received thrombolytic therapy (*n* = 46), had a history of statin therapy before current stroke (*n* = 164), had undergone no statin administration within 72 h from symptom onset (*n* = 111), had first statin administration after the development of END (*n* = 6), and had a change in statin dose within 21 days even without END (*n* = 31) were excluded. Ultimately, 492 patients were included in our study (Figure 1). 

In total, 89 patients (22.8%) received moderate-intensity statins and 403 patients (81.2%) received high-intensity therapy. No patient was treated with a low-intensity statin according to eligibility criteria. The mean age of the included patients was 67.2 ± 12.0 years, and 60.4% were men. The median (interquartile range) NIHSS score on admission was 3.0 (1.0–4.0). The END group was older and had higher NIHSS score on admission and higher incidence of branch atheromatous lesion and use of high-intensity statin. The baseline characteristics of patients according to the development of END are presented in Table 1. In addition, detailed information about the statin type, dose in this study, and baseline characteristics according to statin intensity were investigated, and it is presented in Supplementary Tables S1 and S2.

### 3.2. Predictors of END

END occurred in 102 patients (20.7%). The cause of END was stroke progression (*n* = 99, 97.0%), unknown causes (*n* = 2, 2.0%), and other causes (*n* = 1, 1.0%). The median time from onset to END was 39.0 (23.0–77.0) h. The incidence of END was significantly higher in moderate- to low-intensity statin compared to high-intensity statin irrespective of statin type, including atorvastatin and rosuvastatin (Supplementary Tables S3 and S4). In univariate analysis, older age (OR, 1.03; 95% CI 1.01–1.05; *p* < 0.001), initial NIHSS score (OR, 1.10; 95% CI 1.01–1.19; *p* = 0.03), shorter onset to arrival time (OR, 0.98; 95% CI 0.96–0.99; *p* = 0.005), and branch atheromatous lesion (OR, 3.32; 95% CI 2.13–5.23; *p* < 0.001) were significant. In particular, lower use of high-intensity statin (OR, 0.46; 95% CI 0.28–0.77; *p* < 0.001) was associated with END. In a multivariable logistic regression analysis, older age (aOR, 1.02; 95% CI 1.00–1.05; *p* = 0.017), branch atheromatous lesion (aOR, 3.49; 95% CI 2.16–5.74; *p* < 0.001), and lower use of high-intensity statin (aOR, 0.44; 95% CI 0.25–0.77; *p* = 0.004) remained significant predictors of END (Table 2). In addition, comparison of the effect of high-intensity statin on END among several subgroups revealed a consistent trend in the beneficial effect on the preventing END in the high-intensity statin group Supplementary Figure S1.

For overcoming the imbalance of patients between two groups, sensitivity analysis using propensity score matching was performed. After matching, standard mean difference was overall decreased among various variables and SMD < 0.20 in various variables except the history of antiplatelet and hypertensive medication and the incidence of END, but standard mean difference of these variable was also decreased (Supplementary Tables S5 and S6). In multivariable analysis using matching cohort by propensity score matching, the use of high-intensity statin was associated with the prevention of early neurological deterioration (END) in single subcortical infarction (SSSI) (aOR, 0.39, 95% CI, 0.20–0.75, *p* = 0.004) (Table 3). 

### 3.3. Statin Initiation Time from Symptom Onset

**Figure 2:**
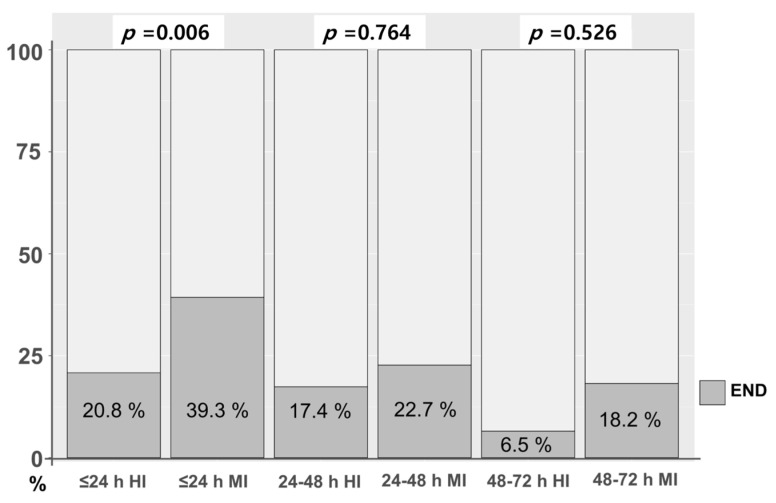
END according to initiation time of statin between high- and moderate-intensity statin. END, early neurologic deterioration; HI, high-intensity statin; MI, moderate-intensity statin.

We examined whether the initiation time of statins (≤24 h, 24–48 h, and 48–72 h) according to statin intensity affected END. There was no significant difference in the initiation time of statin therapy between high- and moderate-intensity statins (*p* = 0.608). However, the incidence of END according to statin intensity differed by initiation time of statin. In patients whose initiation of statin occurred within 24 h, there was a markedly lower incidence of END in patients with high-intensity statin compared to those with moderate-intensity statin (20.8% vs. 39.3%, *p* = 0.006). After 24 h, the incidence of END tended to be lower in patients taking high-intensity statins than in those taking moderate-intensity statins between 24–48 h and 48–72 h. However, the difference was not significant (Figure 2).

### 3.4. Functional Outcome at 3 Months

Of the 492 patients, 488 patients (99.2%) had mRS data available. The rates of favorable functional outcome at 3 months (mRS 0–2) in patients with and without END were 59.4% and 86.3%%, respectively (Supplementary Figure S2). After adjusting for relevant confounders, END was significantly associated with a less favorable functional outcome at 3 months (aOR 0.18, 95% CI, 0.09–0.35, *p* < 0.001). 

## 4. Discussion

The statin dose relationship has been investigated in patients with atherosclerotic cardiovascular disease (ASCVD). According to these trials, ACC/AHA guidelines recommend high-intensity statin therapy for patients with ASCVD [14]. However, evidence is lacking regarding whether high-intensity statins should be administered in cases of lacunar stroke. The major finding of the current study was that high-intensity statin therapy within 72 h from symptom onset might reduce the development of END in patients with SSSI compared to moderate-intensity statin therapy. 

Although intrinsic disease of the penetrating arteries is believed to cause a small subcortical infarction, recent studies have shown that large-artery atherosclerosis is frequently found in small subcortical infarction. One recent study using high-resolution magnetic resonance vessel wall imaging showed that 88.4% of the patients with small subcortical infarction had atherosclerotic plaque in the middle cerebral artery corresponding to infarcted lesion [15]. Therefore, high-intensity statins may be effective in patients with a single small subcortical infarction caused by atherosclerotic plaques.

The development of END is an important prognostic factor for outcome after ischemic stroke, irrespective of stroke etiology [16]. Particularly in SSSI, although the rate of END in lacunar stroke varies according to definition, approximately 20% of patients are known to suffer from END. Moreover, the development of END in SSSI has been shown to increase the risk of poor functional outcomes [17,18,19]. In our study, END occurred in 20.7% of patients and was associated with poor functional outcomes at 3 months after index stroke. Therefore, the prevention of END and the application of appropriate treatment strategies after END cannot be overemphasized for favorable outcomes after SSSI. Nevertheless, there is no established treatment for the prevention and treatment of END. Based on this study’s results, high-intensity statin therapy could be a feasible option for improving outcomes after SSSI by preventing END. 

Several studies have identified potential mechanisms of END in SSSI. The atherosclerotic process is one of the hypothesized mechanisms in the pathogenesis of END in SSSI [5]. Other mechanisms included edema, excitotoxicity, and inflammation [20,21,22]. Consistent with previous studies, the presence of branch atheromatous lesion in SSSI was associated with the development of END in this study. This finding indicated that branch atheromatous lesion might be the main cause of END in SSSI. The preventive effects of statins on atherosclerosis, inflammation, and plaque stabilization are well-known benefits of statin use [23,24,25]. 

The initiation timing of statin after ischemic stroke influenced outcomes in previous studies [26]. Early statin use after hospitalization was associated with greater post-stroke survival [27]. In addition, even in patients who underwent intravenous thrombolysis, the benefit in short- and long-term prognosis was greater when statins were started within 24 h than when they were started within 24 to 72 h [26]. This study demonstrated that high-intensity statin therapy within 72 h was associated with a reduced incidence of END. In particular, the effect might be greater when statins are administrated within 24 h, which is in line with previous studies. Therefore, considering the potential benefits, early treatment with high-intensity statin could be a possible option to prevent the development of END. 

In our study, when statin initiation time was divided from symptom onset, the overall incidence of END was lower in patients who received statin late (Figure 2). This finding did not indicate the benefit for late administration of statin due to the characteristics of END, which decreased incidence over time [10].

One of the strengths of this study is that it included a prospective database and identified END according to a predetermined definition. Another strength is that it included patients with SSSI who had no clear indication for statin therapy and who received statins in the acute phase (≤72 h), a frequently used treatment protocol in real-world practice. Therefore, our results can be applied in clinical practice for patients with ischemic stroke. 

Our study has several limitations. First, it is a retrospective observational study and single-center study. Therefore, a selection bias could not be excluded. Second, we did not use advanced imaging techniques such as vessel wall MRI. Therefore, this study included patients with different SSSI mechanisms. Third, although we collected information about antiplatelet dose in the acute phase, the duration of antiplatelet use was not ascertained. Fourth, we did not assess white matter hyperintensities or cerebral microbleeds that could affect the outcome or recurrence after SSSI [28,29]. Fourth, patients included in this study were limited to Asian ancestry in single hospital. 

## 5. Conclusions

Early administration of high-intensity statins within 72 h was associated with lower rates of END development in patients with SSSI. Furthermore, lower END development in SSSI was significantly associated with favorable outcomes. Therefore, early administration of high-intensity statin therapy within 72 h could be a possible option to improve the outcome of SSSI. However, further randomized clinical trials are needed.

## Figures and Tables

**Table 1:** Baseline characteristics of patients with or without early neurological deterioration.

	Non-END (*n* = 390)	END (*n* = 102)	*p* Value
Age, y, mean ± SD	66.4 ± 11.8	70.3 ± 12.1	0.003
Male, *n* (%)	241 (61.8%)	56 (54.9%)	0.249
NIHSS score on admission, median (IQR)	2.0 [1.0; 4.0]	3.0 [2.0; 5.0]	0.032
Onset to arrival time, h, median (IQR)	16.2 [5.1; 31.2]	11.4 [4.9; 23.3]	0.046
**Vascular risk factors, *n* (%)**			
Hypertension	248 (63.6%)	63 (61.8%)	0.822
Diabetes mellitus	115 (29.5%)	29 (28.4%)	0.931
Dyslipidemia	67 (17.2%)	19 (18.6%)	0.844
Coronary artery disease	9 (2.3%)	4 (3.9%)	0.577
Smoking	170 (43.6%)	41 (40.2%)	0.614
Prior stroke or TIA	36 (9.2%)	12 (11.8%)	0.562
**Laboratory findings, mean ± SD**			
Fasting glucose, mg/dL	117.0 ± 41.4	117.2 ± 40.9	0.952
LDL-C, mg/dL	122.4 ± 36.4	127.7 ± 38.6	0.198
HDL-C, mg/dL	45.7 ± 12.6	45.0 ± 11.4	0.617
Triglyceride, mg/dL	137.6 ± 74.0	132.2 ± 83.4	0.523
Total cholesterol, mg/dL	184.4 ± 41.2	188.0 ± 40.0	0.426
CRP, mg/dL	0.7 ± 1.6	0.7 ± 1.6	0.981
Systolic blood pressure, mm Hg	156.6 ± 26.8	158.8 ± 23.8	0.445
Diastolic blood pressure, mm Hg	89.7 ± 16.3	90.3 ± 14.8	0.708
**Prior medication, *n* (%)**			
Antiplatelet	57 (14.6%)	17 (16.7%)	0.719
Antihypertensive treatment	145 (37.2%)	35 (34.3%)	0.675
**Neuroimaging analysis**			
Branch atheromatous lesion	114 (29.2%)	59 (57.8%)	<0.001
Parent artery stenosis (0–50%)	132 (33.8%)	38 (37.3%)	0.598
Location of SSSI			0.168
Anterior circulation	201 (51.5%)	61 (59.8%)	
Posterior circulation	189 (48.5%)	41 (40.2%)	
**Regimen of antiplatelet in acute phase, *n* (%)**			0.215
No antiplatelet	13 (3.3%)	6 (5.9%)	
Single antiplatelet	157 (40.3%)	33 (32.4%)	
Dual antiplatelet	220 (56.4%)	63 (61.8%)	
**Initiation time of statin from symptom onset**			0.020
≤24 h	221 (56.7%)	71 (69.6%)	
24–48 h	117 (30.0%)	26 (25.5%)	
48–72 h	52 (13.3%)	5 (4.9%)	
**Intensity of statin therapy in acute phase, *n* (%)**			0.004
Moderate intensity	60 (15.4%)	29 (28.4%)	
High intensity	330 (84.6%)	73 (71.6%)	

END, early neurologic deterioration; SD, standard deviation; IQR, interquartile range; NIHSS, National Institutes of Health Stroke Scale; TIA, transient ischemic attack; LDL-C, low-density lipoprotein cholesterol; HDL-C, high-density lipoprotein cholesterol; CRP, c-reactive protein; SSSI, single small subcortical infarction.

**Table 2:** Multivariable logistic regression analysis for predictors of END.

	Crude OR		Adjusted OR	
Variables	OR (95% CI)	*p* Value	OR (95% CI)	*p* Value
Age, years	1.03 (1.01–1.05)	<0.001	1.02 (1.00–1.05)	0.017
Male	0.75 (0.48–1.17)	0.206		
NIHSS score on admission	1.10 (1.01–1.19)	0.030	1.05 (0.96–1.16)	0.263
Onset to arrival time, hours	0.98 (0.96–0.99)	0.005	0.97 (0.94–1.00)	0.116
Hypertension	0.92 (0.59–1.46)	0.734		
Diabetes mellitus	0.95 (0.58–1.53)	0.835		
Dyslipidemia	1.10 (0.61–1.91)	0.732		
Coronary artery disease	1.73 (0.46–5.43)	0.371		
Smoking	0.87 (0.56–1.35)	0.538		
Prior stroke or TIA	1.31 (0.63–2.56)	0.444		
Fasting glucose	1.00 (0.99–1.01)	0.952		
LDL-C	1.00 (0.99–1.01)	0.198	1.00 (0.99–1.01)	0.144
HDL-C	0.99 (0.98–1.01)	0.615		
Triglyceride	1.00 (0.99–1.00)	0.522		
Total cholesterol	1.00 (0.99–1.01)	0.425		
CRP	1.00 (0.86–1.13)	0.981		
Systolic blood pressure	1.00 (0.99–1.01)	0.444		
Diastolic blood pressure	1.00 (0.99–1.02)	0.707		
Prior medication, antiplatelet	1.17 (0.63–2.07)	0.606		
Prior medication, antihypertensive	0.88 (0.55–1.39)	0.593		
Branch atheromatous lesion	3.32 (2.13–5.23)	<0.001	3.49 (2.16–5.74)	<0.001
Parent artery stenosis (0–50%)	1.16 (0.73–1.82)	0.519		
Location of SSSI		0.137		0.375
Anterior circulation (reference) vs. posterior circulation	0.71 (0.46–1.11)		0.80 (0.49–1.30)	
Regimen of antiplatelet in acute phase		0.221		
No antiplatelet	Reference	-		
Single antiplatelet	0.46 (0.17–1.37)	0.137		
Dual antiplatelet	0.62 (0.23–1.83)	0.353		
Initiation time of statin from symptom onset		0.026		0.887
≤24 h	Reference	-	Reference	-
24–48 h	0.69 (0.41–1.13)	0.150	1.01 (0.45–2.35)	0.966
48–72 h	0.30 (0.10–0.71)	0.013	0.75 (0.13–4.17)	0.750
Intensity of statin therapy in acute phase		0.002		0.004
Moderate intensity (reference) vs. high intensity	0.46 (0.28–0.77)		0.44 (0.25–0.77)	

Data are presented as *n* (%), or median [interquartile range]. END, early neurologic deterioration; OR, odds ratio; CI, confidence interval; NIHSS, National Institutes of Health Stroke Scale; TIA, transient ischemic attack; LDL-C, low-density lipoprotein cholesterol; HDL-C, high-density lipoprotein cholesterol; CRP, C-reactive protein; SSSI, single small subcortical infarction.

**Table 3:** Multivariable logistic regression analysis for predictors of END after 2:1 propensity score matching.

	Crude OR		Adjusted OR	
Variables	OR (95% CI)	*p* Value	OR (95% CI)	*p* Value
Age, years	1.03 (1.00–1.06)	0.045	1.02 (0.98–1.05)	0.327
Male	0.89 (0.50–1.60)	0.703		
NIHSS score on admission	1.15 (1.03–1.29)	0.011	1.14 (0.99–1.30)	0.052
Onset to arrival time, hours	0.98 (0.96–0.99)	0.038	0.97 (0.93–1.01)	0.176
Hypertension	0.91 (0.50–1.66)	0.746		
Diabetes mellitus	0.99 (0.51–1.87)	0.980		
Dyslipidemia	1.48 (0.62–3.32)	0.352		
Coronary artery disease	0.90 (0.04–6.22)	0.925		
Smoking	1.03 (0.56–1.85)	0.923		
Prior stroke or TIA	1.03 (0.36–2.55)	0.947		
Fasting glucose	1.00 (0.99–1.01)	0.095	1.00 (1.00–1.01)	0.102
LDL-C	1.00 (0.99–1.01)	0.211		
HDL-C	1.00 (0.98–1.02)	0.950		
Triglyceride	0.99 (0.99–1.00)	0.976		
Total cholesterol	1.00 (0.99–1.01)	0.584		
CRP	1.01 (0.83–1.18)	0.877		
Systolic blood pressure	1.00 (0.99–1.01)	0.462		
Diastolic blood pressure	1.00 (0.99–1.02)	0.331		
Prior medication, antiplatelet	0.73 (0.31–1.54)	0.434		
Prior medication, antihypertensive	0.98 (0.53–1.78)	0.962		
Branch atheromatous lesion	3.26 (1.78–6.01)	<0.001	2.71 (1.35–5.51)	0.005
Parent artery stenosis (0–50%)	1.08 (0.58–1.97)	0.804		
Location of SSSI		0.060		0.099
Anterior circulation (reference) vs. posterior circulation	0.55 (0.29–1.01)		0.55 (0.26–1.10)	
Regimen of antiplatelet in acute phase		0.568		
No antiplatelet	Reference	-		
Single antiplatelet	0.46 (0.14–1.61)	0.197		
Dual antiplatelet	0.49 (0.16–1.70)	0.235		
Initiation time of statin from symptom onset		0.051		0.920
≤24 h	Reference	-	Reference	-
24–48 h	0.74 (0.36–1.43)	0.381	0.96 (0.33–2.81)	0.950
48–72 h	0.31 (0.07–0.93)	0.064	0.88 (0.09–7.28)	0.907
Intensity of statin therapy in acute phase		0.002		0.004
Moderate intensity (reference) vs. high intensity	0.40 (0.22–0.73)		0.39 (0.20–0.75)	

END, early neurologic deterioration; OR, odds ratio; CI, confidence interval; NIHSS, National Institutes of Health Stroke Scale; TIA, transient ischemic attack; LDL-C, low-density lipoprotein cholesterol; HDL-C, high-density lipoprotein cholesterol; SSSI, single small subcortical infarction.

## Data Availability

If required, our data can be submitted.

## Supplementary Materials

The following supporting information can be downloaded at: https://www.mdpi.com/article/10.3390/jcm12093260/s1, Supplementary Methods: The definitions of END causes. Supplementary Table S1: Summary of statin drug and dose use within 72 h of symptom onset; Supplementary Table S2: Baseline characteristics of patients according to statin intensity; Supplementary Table S3. Early neurological deterioration between high-intensity atorvastatin and moderate- to low-intensity atorvastatin; Supplementary Table S4. Early neurological deterioration between high-intensity atorvastatin and moderate- to low-intensity rosuvastatin; Supplementary Table S5. Baseline characteristics before propensity score matching; Supplementary Table S6. Baseline characteristics after propensity score matching; Supplementary Figure S1. Subgroup analysis of END according to statin intensity; Supplementary Figure S2. Functional outcome at 3 months according to END.
