# Supplementary material for: Impact of High-Intensity Statin on Early Neurologic Deterioration in Patients with Single Small Subcortical Infarction
Source: J Clin Med. 2023 May 3;12(9):3260. doi: 10.3390/jcm12093260 (PMC10179331; doi:10.3390/jcm12093260)
Supplement: Supplementary file 1 [file jcm-12-03260-s001.zip › jcm-2309285-supplementary.pdf]

Supplemental Material

Supplementary Methods

The definitions of END causes

Stroke recurrence was defined as neurological deterioration due to discrete new ischemic lesion in patients who were stable for ≥ 24 hours before occurrence of neurological deterioration. If this event was attributed to cerebral edema, mass effect, brain herniation syndrome or hemorrhagic transformation, it is not categorized into stroke recurrence. Stroke progression was defined as neurological deterioration due to progression of initial infarcted lesion (enlargement of initial infarcted lesion, perilesional edema), which was diagnosed on follow up imaging. Symptomatic hemorrhagic transformation was defined as neurological deterioration could be explained by visible hemorrhagic transformation on follow-up imaging. Other cause was defined as neurological deterioration due to probable medical conditions such as pneumonia, urinary tract infection or side effects of medication. Unknown cause was defined as neurological deterioration could not be explained by above END cause.

Supplementary Table S1. Summary of Statin drug and dose use within 72 hours of symptom onset

High-intensity statin (n = 403)	Moderate-intensity statin (n=89)
Atorvastatin 80mg (n = 215)	Atorvastatin 20mg (n = 38)
Atorvastatin 40mg (n = 169)	Atorvastatin 10mg (n = 9)
Rosuvastatin 20mg (n = 19)	Rosuvastatin 10mg (n = 42)

Supplementary Table S2. Baseline characteristics of Patients with or without Early Neurological Deterioration

	**Total (n=492)**	**Moderate-intensity statin (n=89)**	**High-intensity statin (n=403)**	**P value**
Age, y, mean ± SD	67.2 ± 12.0	69.4 ± 10.8	66.7 ± 12.2	0.060
Male, n (%)	297 (60.4%)	49 (55.1%)	248 (61.5%)	0.312
Initial NIHSS score, median (IQR)	3.0 [1.0;4.0]	3.0 [1.0; 4.0]	3.0 [1.0; 4.0]	0.977
Onset to arrival time, h, median (IQR)	14.7 [5.0;28.5]	14.7 [3.7;27.2]	14.6 [5.4;28.9]	0.340
**Vascular risk factors, n (%)**				
Hypertension	311 (63.2%)	53 (59.6%)	258 (64.0%)	0.503
Diabetes mellitus	144 (29.3%)	22 (24.7%)	122 (30.3%)	0.361
Dyslipidemia	86 (17.5%)	7 (7.9%)	79 (19.6%)	0.013
Coronary artery disease	13 (2.6%)	3 (3.4%)	10 (2.5%)	0.914
Smoking	211 (42.9%)	39 (43.8%)	172 (42.7%)	0.938
Prior stroke or TIA	48 (9.8%)	9 (10.1%)	39 (9.7%)	1.000
**Laboratory findings, mean ± SD**				
Fasting glucose, mg/dL	117.0 ± 41.2	109.1 ± 32.5	118.7 ± 42.7	0.023
LDL-C, mg/dL	123.5 ± 36.9	117.8 ± 36.9	124.7 ± 36.8	0.108
HDL-C, mg/dL	45.6 ± 12.3	44.4 ± 9.8	45.8 ± 12.8	0.236
Triglyceride, mg/dL	136.5 ± 76.0	128.1 ± 73.1	138.3 ± 76.6	0.252
Total cholesterol, mg/dL	185.2 ± 40.9	178.6 ± 40.3	186.6 ± 41.0	0.098
CRP, mg/dL	0.7 ± 1.6	0.5 ± 1.4	0.8 ± 1.7	0.065
Systolic blood pressure, mm Hg	157.1 ± 26.2	154.9 ± 26.3	157.5 ± 26.2	0.388
Diastolic blood pressure, mm Hg	89.8 ± 16.0	89.1 ± 14.1	89.9 ± 16.4	0.652
**Prior medication, n (%)**				
Antiplatelet	74 (15.0%)	23 (25.8%)	51 (12.7%)	0.003
Antihypertensive treatment	180 (36.6%)	41 (46.1%)	139 (34.5%)	0.054

**Neuroimaging analysis**				
Branch atheromatous lesion	173 (35.2%)	30 (33.7%)	143 (35.5%)	0.845
Parent artery stenosis (0-50%)	170 (34.6%)	28 (31.5%)	142 (35.2%)	0.579
Location of SSSI				0.095
Anterior circulation	262 (53.3%)	55 (61.8%)	207 (51.4%)	
Posterior circulation	230 (46.7%)	34 (38.2%)	196 (48.6%)	
**Regimen of antiplatelet in acute phase, n (%)**				<0.001
No antiplatelet	19 (3.9%)	6 (6.7%)	13 (3.2%)	
Single antiplatelet	190 (38.6%)	59 (66.3%)	131 (32.5%)	
Dual antiplatelet	283 (57.5%)	24 (27.0%)	259 (64.3%)	
**Initiation time of statin from sy mptom onset**				0.608
≤ 24 hours	292 (59.3%)	56 (62.9%)	236 (58.6%)	
24-48 hours	143 (29.1%)	22 (24.7%)	121 (30.0%)	
48-72 hours	57 (11.6%)	11 (12.4%)	46 (11.4%)	
END, n (%)	102 (20.7%)	29 (32.6%)	73 (18.1%)	0.004

Data are presented as n (%), or median [interquartile range]. SD indicates standard deviation; IQR, interquartile range; END, early neurologic deterioration; NIHSS, National Institutes of Health Stroke Scale; TIA, Transient ischemic attack; LDL-C, low-density lipoprotein cholesterol; HDL-C, high-density lipoprotein cholesterol; CRP, c-reactive protein; SSSI, Single small subcortical infarction; END, early neurological deterioration.

Supplementary Table S3. Early neurological deterioration between high-intensity atorvastatin and moderate to low intensity atorvastatin

	High-intensity (n=384)	Moderate to low intensity (n=48)	P-value
END	70 (18.2)	21 (43.8)	<0.001

Data are presented as n (%). END, early neurological deterioration

Supplementary Table S4. Early neurological deterioration between high-intensity atorvastatin and moderate to low intensity rosuvastatin.

	High-intensity (n=19)	Moderate to low intensity (n=42)	P-value
END	3 (15.8)	16 (38.1)	0.026

Data are presented as n (%). END, early neurological deterioration\

Supplementary Table S5. Baseline characteristics before propensity score matching

	Total (n=492)	Moderate-intensity statin (n=89)	High-intensity statin (n=403)	SMD
Age, y, mean ± SD	67.2 ± 12.0	69.4 ± 10.8	66.7 ± 12.2	0.067
Male, n (%)	297 (60.4%)	49 (55.1%)	248 (61.5%)	0.034
Initial NIHSS score, median (IQR)	3.0 [1.0;4.0]	3.0 [1.0; 4.0]	3.0 [1.0; 4.0]	0.016
Onset to arrival time, h, median (IQR)	14.7 [5.0;28.5]	14.7 [3.7;27.2]	14.6 [5.4;28.9]	0.022
**Vascular risk factors, n (%)**				
Hypertension	311 (63.2%)	53 (59.6%)	258 (64.0%)	0.092
Diabetes mellitus	144 (29.3%)	22 (24.7%)	122 (30.3%)	0.124
Dyslipidemia	86 (17.5%)	7 (7.9%)	79 (19.6%)	0.345
Coronary artery disease	13 (2.6%)	3 (3.4%)	10 (2.5%)	0.053
Smoking	211 (42.9%)	39 (43.8%)	172 (42.7%)	0.023
Prior stroke or TIA	48 (9.8%)	9 (10.1%)	39 (9.7%)	0.015
**Laboratory findings, mean ± SD**				
Fasting glucose, mg/dL	117.0 ± 41.2	109.1 ± 32.5	118.7 ± 42.7	0.089
LDL-C, mg/dL	123.5 ± 36.9	117.8 ± 36.9	124.7 ± 36.8	0.039
HDL-C, mg/dL	45.6 ± 12.3	44.4 ± 9.8	45.8 ± 12.8	0.128
Triglyceride, mg/dL	136.5 ± 76.0	128.1 ± 73.1	138.3 ± 76.6	0.005
Total cholesterol, mg/dL	185.2 ± 40.9	178.6 ± 40.3	186.6 ± 41.0	0.017
CRP, mg/dL	0.7 ± 1.6	0.5 ± 1.4	0.8 ± 1.7	0.115

Systolic blood pressure, mm Hg	157.1 ± 26.2	154.9 ± 26.3	157.5 ± 26.2	0.101
Diastolic blood pressure, mm Hg	89.8 ± 16.0	89.1 ± 14.1	89.9 ± 16.4	0.055
**Prior medication, n (%)**				
Antiplatelet	74 (15.0%)	23 (25.8%)	51 (12.7%)	0.338
Antihypertensive treatment	180 (36.6%)	41 (46.1%)	139 (34.5%)	0.237
**Neuroimaging analysis**				
Branch atheromatous lesion	173 (35.2%)	30 (33.7%)	143 (35.5%)	0.037
Parent artery stenosis (0-50%)	170 (34.6%)	28 (31.5%)	142 (35.2%)	0.080
Location of SSSI				0.211
Anterior circulation	262 (53.3%)	55 (61.8%)	207 (51.4%)	
Posterior circulation	230 (46.7%)	34 (38.2%)	196 (48.6%)	
**Regimen of antiplatelet in acute phase, n (%)**				0.744
No antiplatelet	19 (3.9%)	6 (6.7%)	13 (3.2%)	
Single antiplatelet	190 (38.6%)	59 (66.3%)	131 (32.5%)	
Dual antiplatelet	283 (57.5%)	24 (27.0%)	259 (64.3%)	
**Initiation time of statin from symptom onset**				0.049
≤ 24 hours	292 (59.3%)	56 (62.9%)	236 (58.6%)	
24-48 hours	143 (29.1%)	22 (24.7%)	121 (30.0%)	
48-72 hours	57 (11.6%)	11 (12.4%)	46 (11.4%)	
END, n (%)	102 (20.7%)	29 (32.6%)	73 (18.1%)	0.336

Data are presented as n (%), or median [interquartile range]. SMD, standard mean difference; SD,standard deviation; IQR, interquartile range; END, early neurologic deterioration; NIHSS, National Institutes of Health Stroke Scale; TIA, Transient ischemic attack; LDL-C, low-density lipoprotein cholesterol; HDL-C, high-density lipoprotein cholesterol; CRP, c-reactive protein; SSSI, Single small subcortical infarction; END, early neurological deterioration.

Supplementary Table S6. Baseline characteristics after propensity score matching.

	Total (n=268)	Moderate-intensity statin (n=179)	High-intensity statin (n=89)	SMD
Age, y, mean ± SD	68.9 ± 10.9	68.6 ± 10.9	69.4 ± 10.8	0.067
Male, n (%)	144 (53.9)	95 (53.4)	49 (55.1%)	0.034
Initial NIHSS score, median (IQR)	3 [1-4]	3 [1-4]	2 [1-4]	0.016
Onset to arrival time, h, median (IQR)	18.5 [13.9-26.7]	14.7 [3.4-27.3]	13.4 [4.7-26.4]	0.022
**Vascular risk factors, n (%)**				
Hypertension	166 (62.2)	113 (63.5)	53 (59.6)	0.081
Diabetes mellitus	74 (27.7)	52 (29.2)	22 (24.7)	0.101
Dyslipidemia	32 (12.0)	25 (14.0)	7 (7.9)	0.198
Coronary artery disease	5 (1.9)	2 (1.1)	3 (3.4)	0.151
Smoking	109 (40.8)	70 (39.3)	39 (43.8)	0.091
Prior stroke or TIA	27 (10.1)	18 (10.1)	9 (10.1)	<0.001
**Laboratory findings, mean ± SD**				
Fasting glucose, mg/dL	118.1 ± 84.9	117.4 ± 75.1	119.8 ± 102.9	0.027
LDL-C, mg/dL	120.1 ± 34.6	121.3 ± 33.5	117.8 ± 36.9	0.101
HDL-C, mg/dL	45.5 ± 12.7	46.0 ± 13.9	44.4 ± 9.8	0.138
Triglyceride, mg/dL	133.6 ± 75.0	136.3 ± 75.0	128.1 ± 73.1	0.111
Total cholesterol, mg/dL	182.4 ± 38.0	184.3 ± 36.7	178.6 ± 40.3	0.146
CRP, mg/dL	0.6 ± 1.7	0.7 ± 1.8	0.5 ± 1.4	0.158
Systolic blood pressure, mm Hg	154.9 ± 26.1	154.0 ± 26.2	154.9 ± 26.3	0.001
Diastolic blood pressure, mm Hg	89.0 ± 15.1	88.9 ± 15.6	89.1 ± 14.1	0.012
**Prior medication, n (%)**				
Antiplatelet	51 (19.1)	28 (15.7)	23 (25.8)	0.250
Antihypertensive treatment	102 (38.2)	61 (34.3)	41 (46.1)	0.241
**Neuroimaging analysis**				
Branch atheromatous lesion	78 (29.2)	48 (27.0)	30 (33.7)	0.146
Parent artery stenosis (0-50%)	93 (34.8)	65 (36.5)	28 (31.5)	0.106

Location of SSSI				0.092
Anterior circulation	160 (59.9)	104 (58.4)	54 (62.9)	
Posterior circulation	107 (40.1)	74 (41.6)	33 (37.1)	
**Regimen of antiplatelet in acute phase, n (%)**				0.668
No antiplatelet	14 (5.2)	8 (4.5)	6 (6.7)	
Single antiplatelet	118 (44.2)	59 (33.1)	59 (66.3)	
Dual antiplatelet	135 (50.6)	111 (62.4)	24 (27.0)	
**Initiation time of statin from symptom onset**				0.024
≤ 24 hours	164 (61.4)	108 (60.7)	56 (62.9)	
24-48 hours	71 (26.6)	49 (20.5)	22 (14.7)	
48-72 hours	31 (12.0)	21 (11.8)	11 (12.4)	
END, n (%)	58 (21.7)	29 (16.3)	29 (32.6)	0.384

Data are presented as n (%), or median [interquartile range]. SMD, standard mean difference; SD indicates standard deviation; IQR, interquartile range; END, early neurologic deterioration; NIHSS, National Institutes of Health Stroke Scale; TIA, Transient ischemic attack; LDL-C, low-density lipoprotein cholesterol; HDL-C, high-density lipoprotein cholesterol; CRP, c-reactive protein; SSSI, Single small subcortical infarction; END, early neurological deterioration.

Supplementary Figure S1. Subgroup analysis of END according to statin intensity

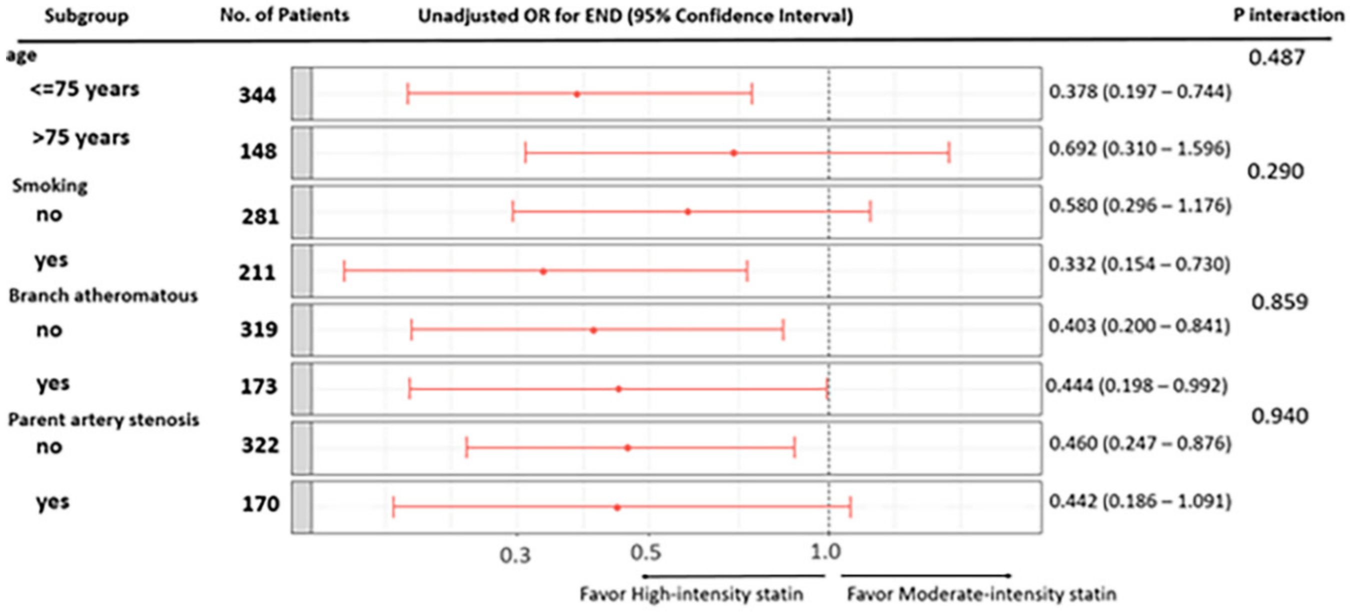

Supplementary Figure S2. Functional outcome at 3 months according to END.

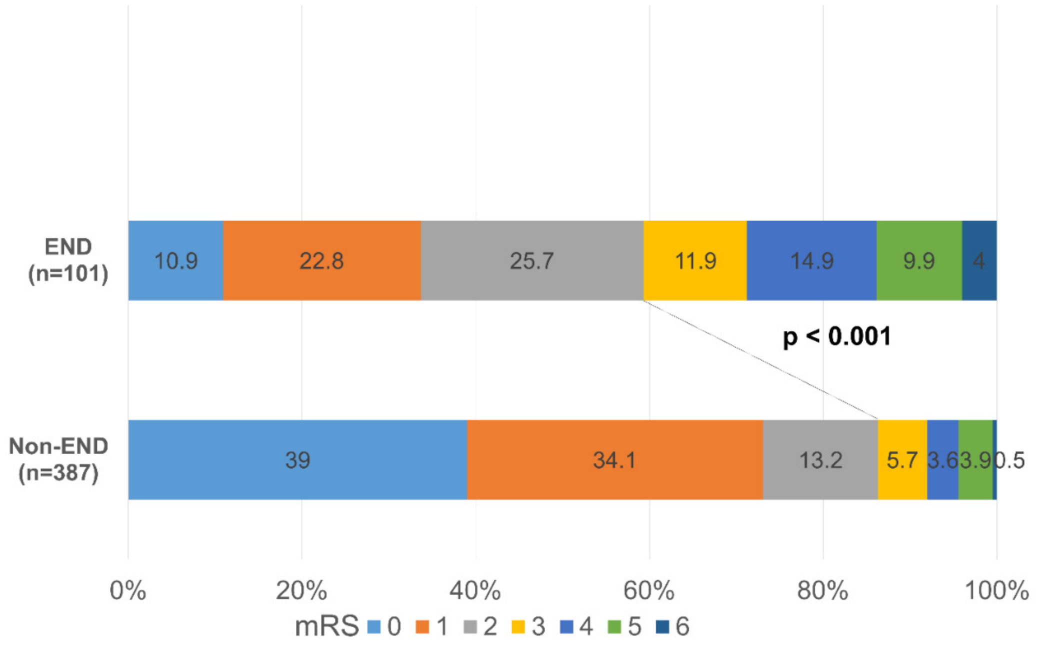

END, early neurologic deterioration; mRS, modified Rankin Scale.
